# Gait Phase Recognition Using Deep Convolutional Neural Network with Inertial Measurement Units

**DOI:** 10.3390/bios10090109

**Published:** 2020-08-27

**Authors:** Binbin Su, Christian Smith, Elena Gutierrez Farewik

**Affiliations:** 1 KTH MoveAbility Lab, Department of Engineering Mechanics, Royal Institute of Technology, 10044 Stockholm, Sweden; binbins@kth.se; 2 KTH BioMEx Center, Royal Institute of Technology, 10044 Stockholm, Sweden; ccs@kth.se; 3 KTH Robotics, Perception and Learning, Royal Institute of Technology, 10044 Stockholm, Sweden; 4 Department of Women’s and Children’s Health, Karolinska Institute, 10044 Stockholm, Sweden

**Keywords:** gait phase recognition, convolutional neural network, IMU

## Abstract

Gait phase recognition is of great importance in the development of assistance-as-needed robotic devices, such as exoskeletons. In order for a powered exoskeleton with phase-based control to determine and provide proper assistance to the wearer during gait, the user’s current gait phase must first be identified accurately. Gait phase recognition can potentially be achieved through input from wearable sensors. Deep convolutional neural networks (DCNN) is a machine learning approach that is widely used in image recognition. User kinematics, measured from inertial measurement unit (IMU) output, can be considered as an ‘image’ since it exhibits some local ‘spatial’ pattern when the sensor data is arranged in sequence. We propose a specialized DCNN to distinguish five phases in a gait cycle, based on IMU data and classified with foot switch information. The DCNN showed approximately 97% accuracy during an offline evaluation of gait phase recognition. Accuracy was highest in the swing phase and lowest in terminal stance.

## 1. Introduction

Exoskeletons are mechanical devices that can help augment a person’s strength or assist movement in people with motor disorders. The use of exoskeletons during rehabilitation can reduce the physical burden of therapists, accommodate data collection during training, and enable a quantitative evaluation of recovery in a controllable manner [1]. The purpose of rehabilitation for patients with lower limb disability is to help them regain lower limb motor function [2]. Gait, or walking, is a cyclic movement exhibiting reoccurring patterns while maintaining static and dynamic balance [3]. A typical gait cycle begins when one foot strikes the ground and ends when the same foot strikes the ground again. A gait cycle can be divided into stance when the foot is in contact with the ground and swing when the limb has no contact with the ground, and further demarcated by gait events, such as ispilateral foot contact, contralateral foot off, etc. Gait phases are defined as durations between consecutive gait events. Accurate identification of gait phases is crucial for metabolically-efficient control of lower limb exoskeletons; inaccurately detected gait phases tend to either increase the user’s effort or exert improper joint torque [4]. When a patient wears an exoskeleton, a gait phase-modulated torque is commonly provided to assist the patient. Martini et al. [5] described hip flexor assistance during swing to reduce the total energy cost of walking with a powered hip exoskeleton called the Active Pelvis Orthosis. Kazerooni et al. [6] divided an entire gait cycle into a loaded stance phase and an unloaded swing phase in the hybrid control of the BLEEX exoskeleton. Position control was used in stance and positive feedback control was applied in swing. Several investigators have focused on a two-phase (stance and swing) classification during one gait cycle [4,7,8,9]. To provide a subtle and continuous control during walking, classifying more specific gait phases is required.

There are numerous methods to identify gait phases, such as camera-based motion capture, force-based systems, and inertial measurement units (IMUs). Most commonly, gait analysis is conducted in a motion analysis laboratory with force platforms and optical motion systems. Such motion capture systems are not easily portable, operate best in controlled environments, and are consequently not optimal to analyze consecutive gait cycles for realistic mobility scenarios. Force-based systems, such as foot switches or force-sensitive resistors, are generally considered the gold standard for detecting gait events, yet they are prone to mechanical failure, unreliable due to weight shifting and provide no details about the swing phase. In the past decade, IMUs have become more common due to their small-size, portability, and high processing power [10,11,12]. Bejarano et al. [13] proposed an adaptive algorithm based on inertial and magnetic sensors to detect gait events. They reported excellent performance from their proposed algorithm in both able-bodied persons and in persons with a gait pathology. Qi et al. [14] sensed foot displacement with IMUs to classify heel strike, heel off, toe off and mid-swing gait phases under different walking speeds, and found no significant variation in recognition accuracy between different walking speeds. Seel et al. [15] proposed a real-time method to detect gait events using accelerometers and gyroscopes. This algorithm was based on setting the exact threshold of foot acceleration and angular velocity for each gait event. They reported that 95% gait events were correctly identified despite the variation between subjects and walking velocity.

Computational methodology has been used to identify gait phases, including threshold-based finite state machine and machine learning approaches [16,17,18]. Threshold-based approaches require laborious work to remove sensor errors and noise, and are inclined to error. These methods depend heavily on crafting the vital features during gait and grows in difficulty when the number of sensors increases [19]. Villarreal et al. [20] expressed difficulty in identifying discrete gait events, especially in the presence of disturbance, in their study of subjects walking on a perturbation platform that randomly moved the stance leg forward or backward. Models have also been proposed to estimate gait phases, such as coupled [21,22] and adaptive oscillators [23], which depend on additional measurement of the system state. Machine learning approaches are data-driven methods that take advantage of a large amount of data and reduce the need to create meaningful features to perform the classification task. Various machine learning approaches, such as K nearest neighbors (KNN) [24], decision trees (DT) [25], Bayesian network classifier (NB) [26], linear discriminant analysis (LDA) [27] etc., have been used for a wide range of gait recognition applications. They can similarly be applied to distinguish gait phases [28]. Bae and Tomizuka [29] proposed a Hidden Markov Model (HMM) to analyse gait phases with the help of ground reaction forces obtained by shoes instructed with air-bladder sensors. Highest accuracy (99%) was achieved in swing and lowest (89%) in terminal stance. Attal et al. [30] presented a Multiple-Regression HMM to automatically segment a gait cycle into six gait phases in an unsupervised manner, and reported the highest overall accuracy of 84%. Deep convolutional neural networks (DCNN) are specialized for processing data that have a grid-like topology, largely applied in feature extraction and image recognition, and have been successfully used to identify human motion and activity from the signal of wearable sensors such as IMUs [31,32,33,34]. A key attribute of the DCNN is its ability to pass the input data through different processing units (e.g., convolution, sigmoid, rectifier and normalization), in contrast to the aforementioned machine learning approaches. The versatile processing units can produce an effective representation of local salience of the signals. Therefore, the features extracted by the DCNN are task-dependent and free from human crafting. Feature extraction and classification are integrated in a single model so their performances are mutually enhanced [33]. Moreover, these features also show more discriminatory power, since the CNN can learn the internal representation through the input-output pairs [35,36]. When the IMU sensor data are put together to form a grid-shape ‘image’ as the input matrix, the DCNN should be able to map the input to an output vector of gait phases, thereby potentially avoiding manually programmed algorithms that capture engineered features of the sensors.

The objectives of this study are to present and evaluate a deterministic machine learning approach DCNN for segmenting gait cycles into five phases based on experimental IMU data in subjects walking in different speeds. The proposed DCNN approach is primarily introduced to analyze the performance on single time frames, which is simpler to implement as an online classifier. The DCNN model can also be a baseline for further comparison if the sequential aspect is considered in the future.

## 2. Experiment Setup and Methods

A convenience example of 12 able-bodied subjects (6 males and 6 females) between 25 and 30 years old were recruited among students and colleagues. Each subject was asked to walk on a treadmill at five different speeds that correspond to mean, mean ±1 standard deviation (SD), and mean ±2 SDs, based on reported comfortable walking speeds normalized to subjects’ ages and height [37]. For men between 20 and 30 years, the mean height-normalized comfortable gait speed and SD are 2840 h${}^{-1}$ and 330 h${}^{-1}$ respectively. For women between 20 and 30 years, the mean height-normalized comfortable gait speed and SD are 3080 h${}^{-1}$ and 350 h${}^{-1}$ respectively. Data were collected for 300 s for each walking speed, for a total of between 1500 and 1700 gait cycles per person. The order of the speeds were randomized in data collection.

Subjects were equipped with seven IMUs (Myon/Cometa aktos-T), attached with tape to the thighs, shanks, feet and pelvis, as well as with foot switches. The foot switches are piezoresistive sensors, and are taped to the sole of the foot at positions that allow a precise measurement of foot contact, support, heel off and toe off phases. They are primarily used to detect gait cycle events. Data was collected at 2000 Hz and down-sampled to 50 Hz to save computational power. Data collection for this study was approved by the Swedish Ethical Review Authority (Dnr. 2020-02311). All subjects gave informed written consent to participate.

**Figure 1:**
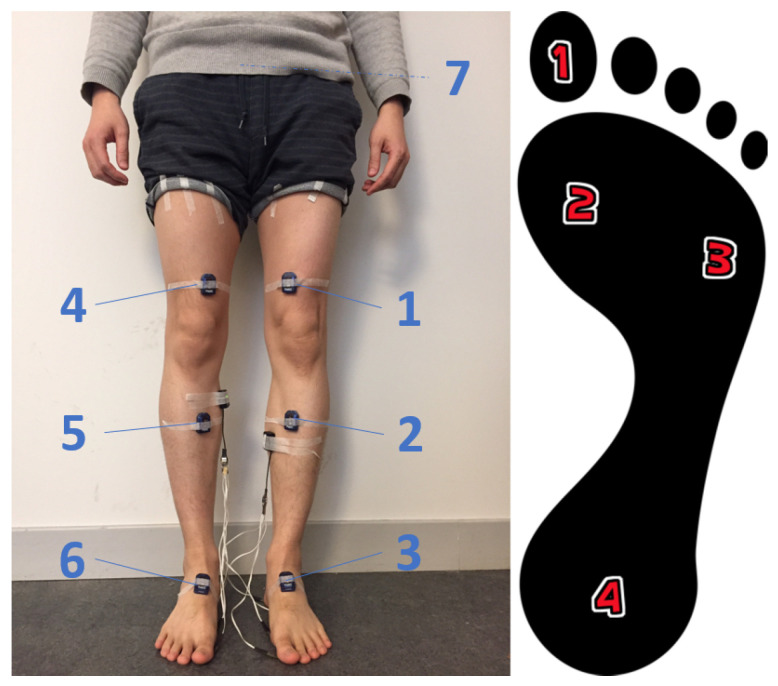
Placement of IMU sensors and foot switches.

Each IMU sensor can record acceleration, angular velocity and magnetic field intensity on three orthogonal axes, for a total of 63 IMU channels. The signal noise of the IMU based measurements may be dependent on the placement and the interconnection to the human body. We placed the IMU inside of a pocket on straps on top of the thighs, shanks, feet and pelvis according to the recommendation from the hardware company (Figure 1). The foot switches were placed under the sole of the feet to detect contact events. Each foot switch consists of four piezoresistive sensors which were placed under the hallux, the first metatarsus, the fifth metatarsus and the heel (Figure 1). The sensors registered contact when pressure was applied, specifically when each sensor returned voltage values of 2.13 V, 1.07 V, 0.53 V, 0.27 V, respectively. The value of a foot switch (VFS) is the sum of its 4 force sensors’ voltages and ranged from 0 V (swing) to 4 V (whole foot contact).

**Figure 2:**
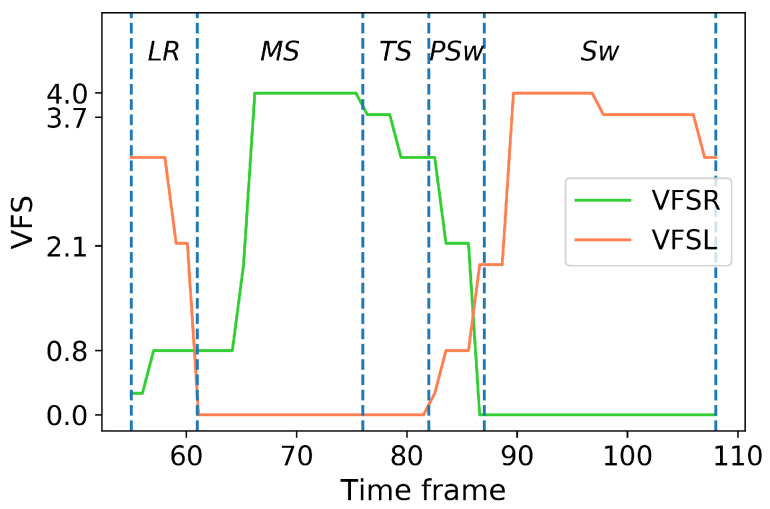
An example of gait phase labeling from VFSR and VFSL in a right gait cycle.

**Figure 3:**
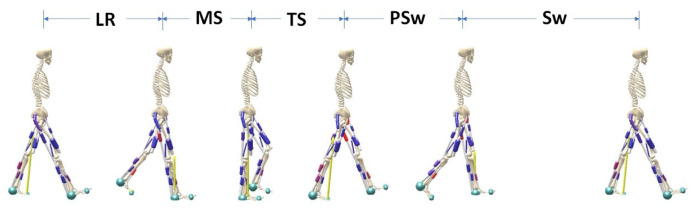
A gait schematic describing 5 gait phases for the right side: loading response (LR), mid-stance (MS), terminal stance (TS), pre-swing (PSw), and swing (Sw). This figure was generated in the open-source software SCONE [39].

The labeling procedure of each gait phase was implemented by defining the phases detected by foot switches as the “ground truth” for 5 gait phases: loading response (LR), midstance (MS), terminal stance (TS), pre-swing (PSw) and swing (SW). The 5 gait phases were defined according to Gage et al. [38] and labelled with left and right VFS (VFSL and VFSR) as (described here for the right side): (Figure 2 and Figure 3):

1. Loading response: begins at ipsilateral foot contact and ends at contralateral foot-off.
  - VFSL>VFSR but VFSR≠0.
2. Mid-stance: begins at contralateral foot off and ends at ipsilateral heel rise.
  - VFSL=0 and VFSR=4V.
3. Terminal stance: begins at ipsilateral heel rise and ends at contralateral foot contact.
  - VFSL=0 and VFSR=3.73V.
4. Pre-swing: begins at contralateral foot contact and ends at ipsilateral foot off.
  - VFSL≠0 and VFSL<VFSR.
5. Swing: begins at ipsilateral foot off and ends at ipsilateral foot contact.
  - VFSR=0.

Acquired IMU time series from each subject were concatenated into an i×j×k matrix, where *i* = number of time frames, *j* = number of IMUs, and *k* = number of IMU channels, in effect transforming the data into a j×k ‘image’ for each frame. In the present study, *j* is always 7 and *k* is always 9. The time period of two adjacent time frames during the testing is 0.02 s.

## 3. DCNN Architechture

### 3.1. Deep Convolutional Neural Networks (DCNN)

DCNNs have been largely applied in the task of feature extraction, image recognition and time series data classification due to the advance of computational power and large amounts of labelled data.

DCNNs can be considered as feed-forward networks where information flows only in one direction from the input to the output. In a DCNN, the input, commonly a three-dimensional tensor, goes through a sequence of processing, usually called a layer. This can be a convolution layer, a pooling layer, a normalization layer, a fully connected layer, a loss layer, etc. [40].

**Figure 4:**
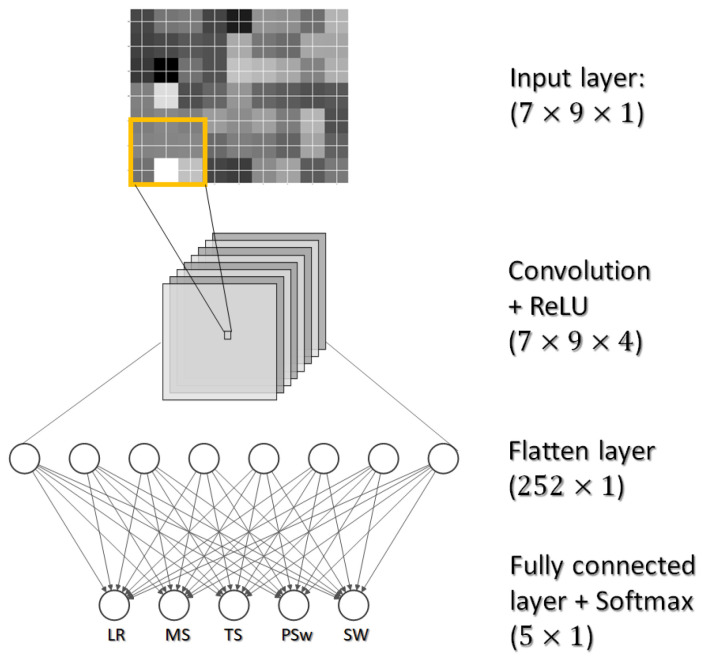
An illustration of DCNN structure classifying IMU “image” into gait phase.

Ideally the DCNN can extract prominent feature from the IMU ‘image’ by applying filters in the convolution layer. After the convolution, the fully connected layers performs the classification task of gait phase detection. In the current study, the DCNN can be seen as a black box with an IMU “image” input on the top and a matching gait phase on the bottom (Figure 4).

The input size of the DCNN is 7×9×1. A convolution kernel is an order 3 tensor with size H×W×D. The input and the convolution kernel always have the same depth. The kernel chosen in our DCNN has a size of 3×3×1. When the convolution layer is applied, the kernel slides over all spatial locations in the input ‘image’, outputting one number at each location by computing dot product between the kernel and a H×W×D chunk of the input ‘image’. In our model, the filter convolves the input ‘image’ by shifting one unit at a time, and no padding is used, the input ‘image’ is padded with zeros around the border and maintained the same size after convolution. By applying multiple convolution layers, we can get multiple response maps. In our model, we have four convolution layers therefore the output *S* after the convolution has a size of 7×9×4.

A Rectified Linear Unit (ReLU) activation was included in the convolution layer in our DCNN to capture the nonlinear relationship of the features. A ReLU layer does not change the size of the input, which means *S* and *R* have the same size (7×9×4).

(1) $$R^{\left(N\right)}=max\left(0,S^{\left(N\right)}\right)$$

After a convolution layer with the ReLU activation, we introduced a flatten layer which removed all dimension except for one. In our case, the input size of the flatten layer was 7×9×4 whose size became 252 after the flatten. The output of the flatten layer contains distributed representations for the input image, and all these features in the current layer can be used to build features with stronger capabilities in the next layers. We then introduced a fully connected layer that gives the final probabilities for each gait phase. The number of nodes in the fully connected layer is 5, which matches the number of the predicted gait phases, therefore the output *s* is an array of 5. To transform the values in *s* to probability *p*, we used a Softmatrix activation (Equation (2)) which maps non-normalized output to the probability distribution over predicted classes.

(2) $$p=\frac{e^{s_{i}}}{\sum_{j}e^{s_{j}}}$$

Finally the predicted class is assigned to the label with the highest probability:

(3) $$k^{*}=arg\max_{1\leq k\leq K}\left\{p_{1},\ldots ,p_{K}\right\}$$

**Figure 5:**
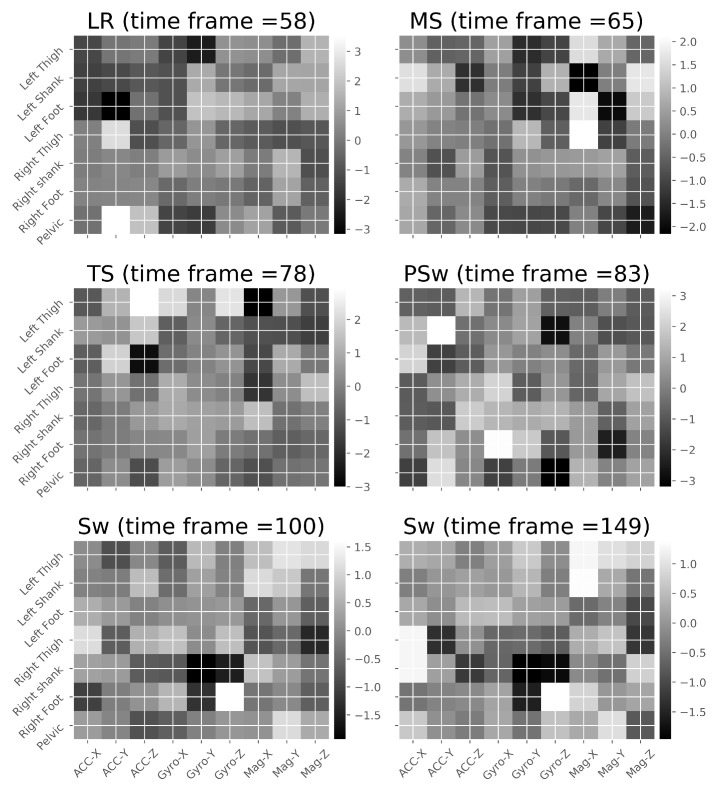
Images representing the IMU features at 6 different time frames, during 5 gait phases.

The input with respect to this study is generated from the seven IMUs and nine channels, which form a 7×9 matrix. The data have been normalized by the mean and standard deviation (Z score normalization, i.e., each value minus the mean, then divided by the standard deviation) of each channel. The row order follows the IMU number from 1 to 7, corresponding to their predefined order in the software. The column order of acceleration, angular velocity and magnetic intensity field corresponds to the order of the raw data. The order of the rows and columns is thus only a convention and does not indicate any rationality. As this convention is adopted for all frames, individual variables will exhibit similarity for the same phase. As the size of ‘image’ can be considered small, we preliminarily tested the use of only an FC layer without a convolution layer. After early observations of poor prediction accuracy of the TS phase, a convolution layer was then added. Example IMU frames from five gait phases are illustrated in Figure 5; the nine IMU channels (acceleration, angular velocity and magnetic field intensity in three directions) are shown on the *x*-axis, and the seven IMUs are shown on the *y*-axis. The raw data thus can be visualised as an ‘image’ at each point in time. The discrepancy of images corresponding to different gait phases can be readily identified while the similarity of the same phase at different time moments (Sw phase at time frames 100 and 149) cannot be omitted.

### 3.2. DCNN Performance Evaluation

The DCNN was implemented three different ways:

1. Intra-subject I: trained the first 70% gait data, and tested on the last 30% data for each speed individually for each subject. This implementation was designed to investigate whether the walking speed affects the recognition accuracy of the gait phases.
2. Intra-subject II: pooled data of all five speeds, then randomly trained on 70% data and tested on the remaining 30% data for each subject individually. This implementation was designed to evaluate the performance of the proposed DCNN classifier when trained with pooled data.
3. Inter-subject: pooled data of all five speeds and all subjects but one, tested on the remaining subject, then iterated for each subject. The training data were measurements from 11 subjects, and test data were from one “unseen” subject, i.e., not included in the training data. This implementation was designed to investigate the reliability of a general model.

Accuracy was computed for each implementation as the proportion of correctly classified phases, i.e., classified phases that matched the true phases. Instead of presenting the F1 scores which usually reflects the false positive (FP) and false negative (FN) rates in binary classification, confusion matrices were introduced to attain FP and FN rates directly for representative subjects.

### 3.3. Other Machine Learning Approaches

The DCNN architecture was trained and tested on a laptop computer (Thinkpad T470p) with an Intel i7-7700HQ CPU @ 2.8GHz and 8GB RAM. The DCNN’s hyperparameters were determined with the grid search approach, including number of batch size, layers and cells. The optimal model was then trained for 100 epochs with an early stop if the model performance did not increase in 10 consecutive epochs, and performance was evaluated on the test set using accuracy. The proposed DCNN in the inter-subject implementation was then compared to some conventional machine learning approaches that are common for solving classification problems, as stated in [41]:

1. K nearest neighbours (KNN) is a non-parametric method that is classified by the majority vote of its neighbors. Among its *k* nearest neighbors, the object is classified as the most common class.
2. Decision tree (DT) is a way to visually represent and make decisions. The tree is constructed by choosing the best question and splitting the input data into subsets. It terminates with unique class label leaves.
3. Naive Bayes (NB) assumes that all features are independent of each other according to Bayes’ theorem. First, the NB classifier creates a probabilistic model that estimates the probability that an input sample belongs to a certain class. For biomechanical gait data, the probabilistic model is commonly implemented by means of a normal distribution. Then, a decision rule is applied to attribute the data to the most likely class.
4. Linear Discriminant Analysis (LDA) projects all data examples on a line by lowering the dimension of the dataset. Then the examples are classified into classes based on the their distance from a chosen point or centroid.

Since the source code and hyperparameters for the classifier are not well revealed, the above conventional classifiers were implemented using standard packages from Scikit-learn, a widely-used machine learning library, with adjusted parameters to maximize the performance. In the case of KNN, the number of neighbours was set to 3, after preliminarily testing between 1 and 10 neighbors. In the case of DT, the default configuration was adopted. For NB, the Gaussian Naive Bayes algorithm was chosen for classification. For LDA, We chose a least squares solution combined with automatic shrinkage using the Ledoit–Wolf lemma.

## 4. Results

For intra-subject I implementation, the computational time for training and testing was approximately 21 s and 0.12 s, respectively, for each speed and each subject. For intra-subject II implementation, the computational time for training and testing was approximately 101 s and 0.34 s, respectively, for each subject. For inter-subject implementation, the computational time for training and testing was approximately 300 s and 0.54 s, respectively. For each implementation, recognition accuracy is showed in the Figures 7–9 as boxplots of median and interquartile range box (IQR).

### 4.1. Intra-Subject I Implementation

**Figure 6:**
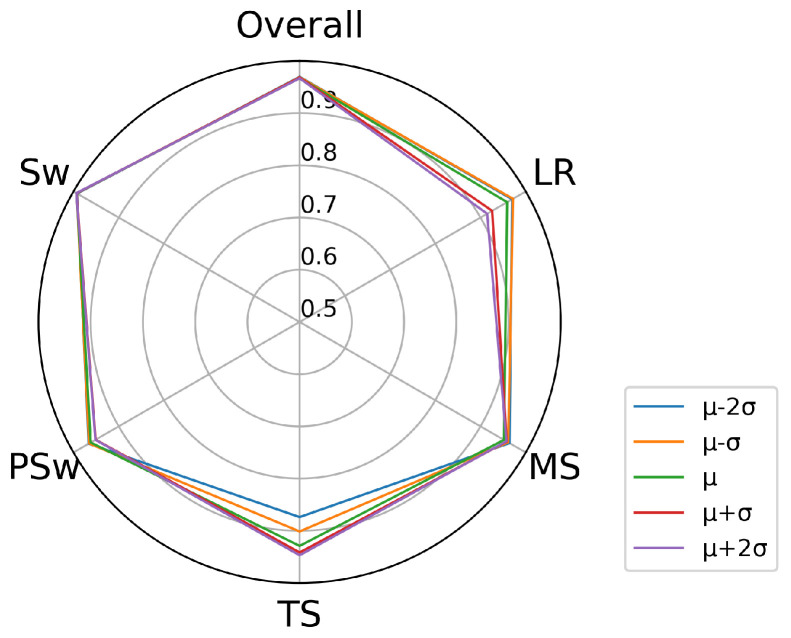
Gait phase recognition accuracy for each phase and overall. The five walking speeds are illustrated separately.

**Figure 7:**
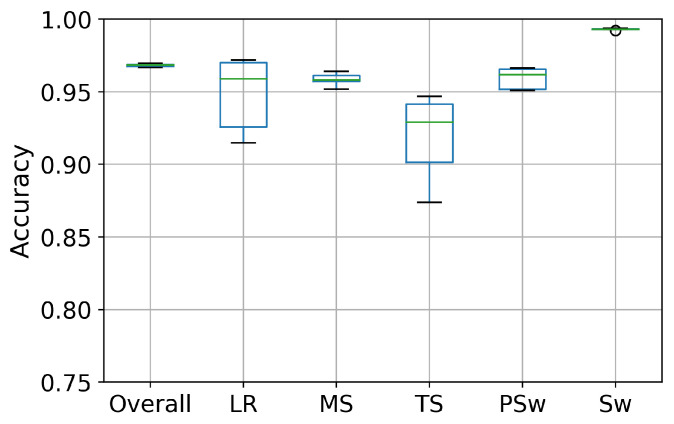
Boxplot displaying the recognition accuracy distribution of five phases and the overall accuracy in the intra-subject I implementation. The line inside the box represents the median accuracy. The box represents the middle 50% of the accuracy. The whiskers represent the ranges for the bottom 25% and the top 25%, and bullets indicate outliers.

**Figure A1:**
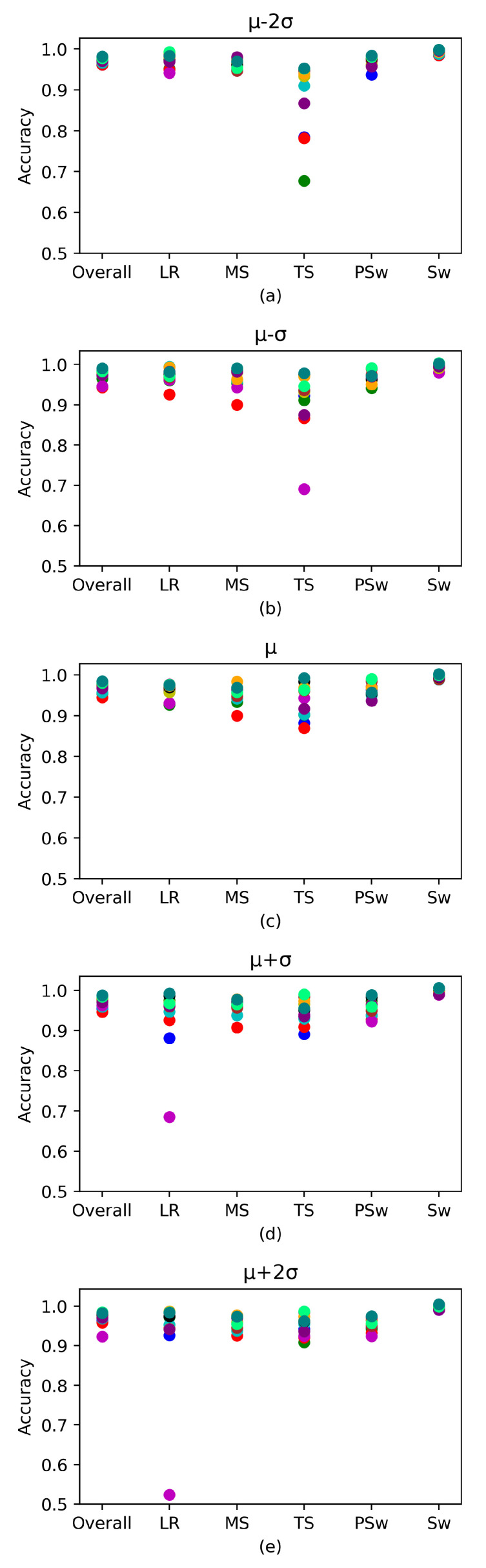
Phase recognition accuracy overall and in the 5 phases, shown for each individual at a walking speed of (**a**) μ− 2σ, (**b**) μ−σ, (**c**) μ, (**d**) μ + σ, (**e**) μ + 2σ.

Gait phase recognition accuracy was computed for five walking speeds separately (Figure 6) as well as together. Overall, the DCNN detected Sw with the highest classification accuracy (99.3%), followed by PSw (96.2%), MS (95.8%) and LR (95.8%). The lowest recognition accuracy was observed for TS (92.9%). Walking speed had a very small influence on recognition accuracy of LR and TS (Figure 6, Appendix A Figure A1), wherein recognition of TS was slightly lower in the slowest (87.3%) than in the mean (92.9%) walking speed. In contrast, recognition of LR was somewhat lower in the fastest (91.4%) than in the mean (95.8%) walking speed. The overall accuracy on all gait phases is approximately 96.8%. The recognition variability was higher in LR and TS than in MS, PSw or Sw (Figure 7).

### 4.2. Intra-Subject II Implementation

**Figure 8:**
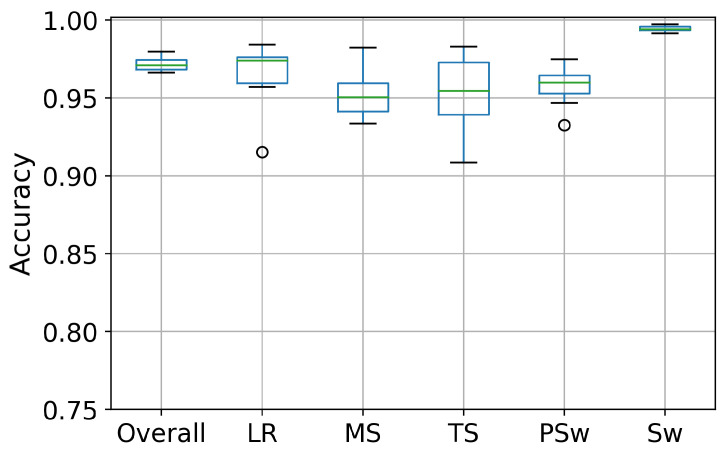
Boxplot displaying the recognition accuracy distribution of five phases and the overall accuracy in the intra-subject II implementation. The line inside the box represents the median accuracy. The box represents the middle 50% of the accuracy. The whiskers represent the ranges for the bottom 25% and the top 25%, and bullets indicate outliers.

When data from all speeds were pooled for each subject, the DCNN again most accurately detected Sw (99.4%), followed by LR (97.3%), PSw (95.9%), TS (95.4%), and MS (95.1%), for an overall accuracy of 97.1%. Recognition variability was highest in LR, TS and PSw (Figure 8).

### 4.3. Inter-Subject Implementation

**Figure 9:**
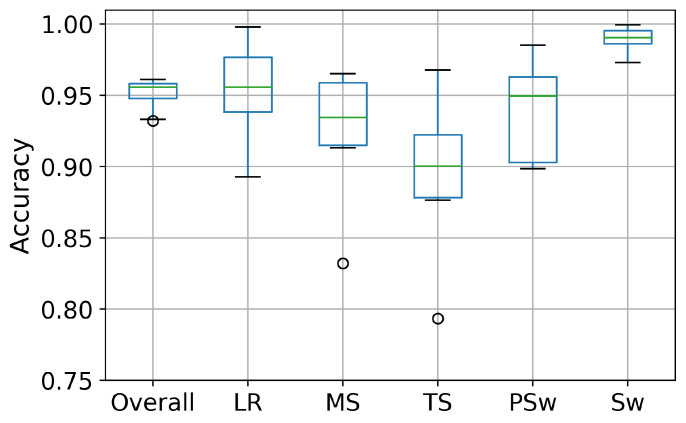
Boxplot displaying the recognition accuracy distribution of five phases and the overall accuracy in the inter-subject implementation. The line inside the box represents the median accuracy. The box represents the middle 50% of the accuracy. The whiskers represent the ranges for the bottom 25% and the top 25% of the accuracy, excluding outliers. The bullets indicate the outliers.

When data from speeds and subjects were pooled, the DCNN again most accurately detected SW (99.0%), followed by LR (95.5%), PS (95.0%), MS (93.4%) and TS (90.0%), for an overall accuracy of 95.6% (Figure 9).

**Figure 10:**
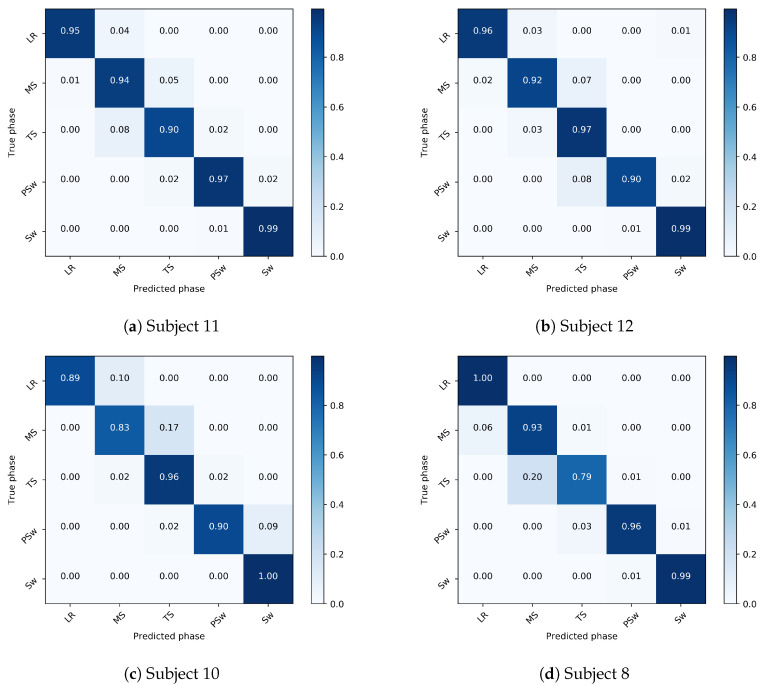
Confusion matrices showing the overall recognition accuracy in 4 example subjects in the inter-subject implementation. The true gait phase is shown on the *y*-axis and the predicted phase is on the *x*-axis. A strong diagonal indicates very accurate recognition, and misclassifications are indicated off the diagonal. The highest accuracy was seen in Subjects 11 and 12 (over 90% accuracy), and lowest in Subject 10 and 8, in whom MS was detected with 83% and TS, with 79% accuracy, respectively. These two subjects are the outliers in Figure 9. In general, it can be observed that when phase detection was incorrect, the DCNN often misclassified a gait phase as adjacent to the true phase.

Several confusion matrices are provided to illustrate subjects in whom the best and worst overall recognition accuracy were observed (Figure 10). It can be seen here that when misclassification did occur, it was typically between adjacent gait phases.

### 4.4. Other Machine Learning Approaches

In the inter-subject implementation, among traditional classifiers the DCNN had the highest recognition accuracy (Table 1). It particularly outperformed the other classifiers in detecting TS. Recognition accuracy was next best with LDA and lowest with the NB.

## 5. Discussion

The proposed DCNN network can accurately and effectively identify gait phases with IMU data fixed to the lower body of able-bodied subjects; approximately 5000 ‘images’ can be classified per second. Although gait is a periodic movement with a repetitive nature and temporal pattern, we explored in this study the treatment of gait phases as discrete events in which strong correlations may exist among measured sensor data within the same gait phases, as well as large difference between different gait phases. The DCNN has been widely used for image classification which means it can also capture the features of the IMU ‘images’ by recognizing the similarity of the same phases and distinguishing the discrepancy of different gait phases. Using different signals (acceleration, angular velocity, magnetic field intensity individually as well as combined) as the feature ‘images’ results in different recognition accuracy of the gait phases, wherein the combined signals presented the best performance in Appendix A Table A1. It is reasonable to infer that the combined signals represent a more comprehensive picture of gait characteristics.

The DCNN was trained by raw IMU ‘images,’ each containing a label that specified which to which gait phase it belongs. The advantage of applying the DCNN approach on raw IMU data instead of first transforming it into 3D joint angles is the time lag potentially avoided from such computations. In the study by Jiang et al. [42], labelling of gait phases was performed by experienced lab engineers or physiotherapists. While that approach has it merits, it is more time-consuming and subjective than an automated approach. In the current study, labelling of gait phases was performed automatically from foot switch data, in which hundreds of gait cycles can be labelled practically instantaneously.

**Figure 11:**
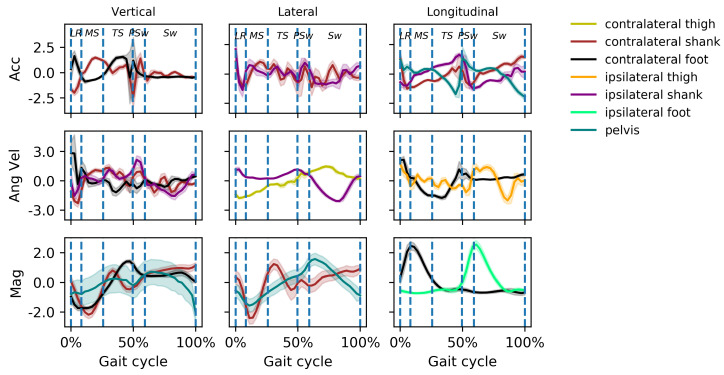
Z-score normalized raw IMU data from a representative subject (Subject 11) at a speed of 6.4 km/h, from IMUs. Mean ±1 SD acceleration (Acc), angular velocity (Ang Vel), and magnetic field intensity (Mag) in the three directions are illustrated for a selection of the 63 available channels. The detected gait phases are indicated. Some characteristics, namely local maxima/minima and inflection points of the raw data coincide with phase transitions.

The properties in the DCNN’s structure that describe what it will “learn” during training are called hyperparameters. The hyperparameters of the DCNN network such as the kernel in the convolution layers, weight matrix, and bias in the fully connected layer were initially randomly generated, thus the DCNN approach was unlikely to accurately predict the gait phases before training. During the training process, these tunable parameters were updated periodically to minimize the loss between predicted gait phases and true phases until the desired classification accuracy was achieved. Although we cannot observe the internal process by which the DCNN learns to identify the corresponding gait phases through IMU ‘images’, it most likely captures the inner feature representation of linear acceleration, angular velocity and heading reference between different gait phases. Figure 11 illustrates normalized raw IMU data from a representative subject at one walking speed. Some characteristics, namely local maxima/minima and inflection points of the the raw data coincide with phase transitions. The magnetic field intensity, for instance, can be characterized by local maxima that coordinate well with the phase changes to PSw and to Sw. These relationships were not specifically coded inside the DCNN network; this approach creates feature maps to identify gait phases from the acceleration, angular velocity and magnetic field intensity data of IMUs, and thus likely uses such changes in gait phase detection.

Overall, the accuracy varied among gait phases in all implementations. Walking speed did not have a major effect on the overall accuracy of gait phase recognition for people with a typical gait pattern. However, phase recognition of Sw was overall the most accurate, and of TS overall, the least accurate, even more so at lower speeds. TS is the last sub-phase of single support during which the center of mass advances over the foot until double support. The definition of TS used in the current study, as per Gage et al. [38], is that TS begins at heel rise and ends at contralateral foot contact. The relatively low recognition accuracy of this phase can be attributed to the observation that there are few characteristic acceleration or angular velocity changes during TS (Figure 11) probably leading to ambiguity for the DCNN classifier. This ambiguity in distinguishing TS from MS can be seen in Figure 10. In lower walking speed the boundary between TS and MS may be blurred. Our results were consistent with the study by Hebenstreit et al. who reported that walking speed affects the durations of gait sub-phases. They reported particularly high speed dependence in MS and TS, wherein TS duration decreases and MS increases in slower walking speeds [43]. Perry and Davids suggested MS is normally between 12% and 31% and TS is between 31% and 50% of the gait cycle [44]. Our findings showed a longer MS (an average of 12–35% at average speed) and shorter TS (35–47%). This discrepancy might be due to the method by which heel-off is detected, which is supported by [43], who used a heel velocity threshold perpendicular to the treadmill plane to perform event detection. Normally a gait cycle begins as the heel strikes the ground. The whole foot then rotates about the heel. Next the foot remains flat and the tibia rotates over the foot. Then as the fulcrum of the pivot moves anteriorly to the metatarsal heads, the foot rotates about the forefoot. Perry et al. [44] referred these phases as the heel rocker, ankle rocker and forefoot rocker. Gage et al. [38] have described these phases as coinciding with LR, MS and TS, respectively, and we have adopted this this terminology to define gait phases. However, the rockers may not be mutually independent; some overlap may exist between them. For example, the heel rocker starts when the heel strikes the ground and ends with flat foot which constitutes 8% of the gait cycle. The ankle rocker should begin from the minimum dorsiflexion which normally occurs at around 5% of the gait cycle. There is a period in which heel rocker and ankle rocker overlap. Similar overlap is observed between other gait phases, such as MS and TS. There is thus some ambiguity between LR and MS and between MS and TS, to which we can attribute the DCNN’s lower accuracy in detecting LR and TS, particularly when trained and tested on different people. Despite the misclassifications of MS and TS, the overall accuracy in the current study is still higher than that reported earlier [45,46,47]. Unlike an approach that directly specifies a precise threshold of foot acceleration and angular velocity for each gait event [15], the DCNN approach can potentially capture the internal representation of the feature images to perform phase recognition.

For the intra-subject I implementation, the model was trained and tested at each speed separately. It displays high accuracy (96.8% overall), suggesting that the intra-individual variability at a constant walking speed is small, and that the performance of the DCNN is not specifically dependent on walking speed. By pooling the data of all speeds for each subject in the intra-subject II implementation, the overall recognition accuracy was also high (97.1% overall), which suggests that the characteristics in individual IMU channels are still present at different walking speeds. The intra-subject implementations may introduce overfitting as they exploits the gait characteristics in the training subjects’ data, and may thus tend to cause more error in unseen subjects (e.g., in inter-subject implementation). However, the intra-subject implementations support a flexible application of a generic DCNN model trained at collected IMU data at varying walking speeds, rather than necessarily training multiple models for each walking velocity. This also argues for the robustness of the DCNN model since people generally walk at varying speeds throughout the day. A generic model can potentially be formed not only in an experimental environment but also in realistic conditions.

For the inter-subject validation, the DCNN achieved an overall accuracy of 95.1%, which is similar to the performance in the two intra-subject implementations. The accuracy in predicting Sw was higher than in any other phase, in all three implementations. While it is difficult to know the exact reason due to the hidden layers, the main features during Sw were easier for the algorithm to distinguish. One likely factor is the rapid change in foot angular velocity as the foot transitions from stance to swing. This finding is consistent with the results found by [4]. In our data, a rapid change in the foot’s magnetic field intensity can be observed during the transition between PSw and Sw (Figure 11). The accuracy in MS and TS, however, was much lower than that in intra-subject evaluations. This can be attributed to the inherent variation of natural gait patterns between individuals. This finding supports to some extent the concept that gait can seen as a biometric identity in some research applications [48]. The inter-subject variability is large enough that a small subject group (*n* = 12) might not be adequate to generate a general model.

Several other machine learning algorithms achieved a high recognition accuracy. The NB and the KNN both predicted LR and PSw slightly better than the DCNN, but the overall accuracy was highest in the DCNN. KNN measures the mathematical distance of the IMU data points between different gait phases to find similarities. These distances, however, seems to be small during the transition between gait phases. DT ‘prunes’ the tree by selecting the best attributes to make classifications, but no particular attributes are well known for identifying gait phases. NB had the lowest overall accuracy (90.6%) among all classifiers. NB is based on probability theory which is under the assumption that independent and identically distributed (i.i.d.) data are used. However IMU data are time series data, which may violate the i.i.d. assumption. LDA achieved the best overall accuracy (92.6%) among the four traditional classifiers. LDA is a linear classifier which is believed to outperform DT when data has numerours features.

There are some limitations in the current study to consider. The gait phase recognition approach is based on discrete samples extracted from continuous time series data, which does not take into account the time dimension in the continuous data, namely the time dimension in continuous data. Furthermore, the current DCNN model only considers the data in the current time frame to classify the corresponding gait phase; a model that includes data from previous time frames may detect with even higher accuracy. The true gait phases were labelled through foot switch measurement, which may potentially induce errors due to mechanical failure or false activation. Foot switches provide satisfactory results in identifying gait phases for normal walking, but sensor attachment proves difficult when assessing amputees or subjects with foot deformity [49]. Another system using insoles with a matrix of pressure sensors has been reported to have a high reliability in evaluating gait phase durations in normal gait, which could be an alternative to increase the applicability in pathological gait [50]. In addition, it is possible that similar phase recognition accuracy could be achieved with fewer IMUs. Furthermore, the approach in this study was only evaluated on able-bodied, relatively young adults. The achieved results and conclusions might not extrapolate to other age groups and people with disability. Finally, our analyses were performed offline, whereas gait phase detection must be performed in real-time for any applications in exoskeletons or rehabilitation. Future work should thus focus on determining the minimum number of IMUs required, should investigate the possibility of incorporating past and current time frames in the model, and should focus on detecting gait phase in real-time, and in relevant patient populations.

## 6. Conclusions

The presented DCNN model can recognize five gait phases from raw data of seven IMUs attached to the pelvic, thighs, shanks and feet. The proposed model can capture the inner kinematic representation of linear acceleration, rotational velocity and IMU magnetic field between different gait phases, and achieve an overall recognition accuracy of 97.5% on a well-trained model, with up to 99.6% accuracy in detecting the swing phase. This model focuses on a sample-based gait phase recognition approach. Generally, the DCNN performed better when trained and tested on the same individual than when trained on some individuals and tested on others.

## Figures and Tables

**Table 1:** Gait phase recognition accuracy for the deep convolutional neural networks (DCNN), as well as for other traditional classifiers: K nearest neighbours (KNN), Decision tree (DT), Naive Bayes (NB) and Linear Discriminant Analysis (LDA).

Classifiers	Overall	LR	MS	TS	PSw	Sw
DCNN	0.951	0.952	0.927	0.898	0.940	0.990
KNN	0.910	0.947	0.877	0.745	0.957	0.986
DT	0.910	0.929	0.914	0.744	0.930	0.980
NB	0.906	0.962	0.905	0.713	0.993	0.966
LDA	0.926	0.960	0.895	0.797	0.977	0.980

## Abbreviations

The following abbreviations are used in this manuscript:

DCNN	Deep convolutional neural networks
IMU	Inertial measurement unit
VFS	Value of a foot switch
VFSL	Value of the left foot switch
VFSR	Value of the right foot switch
LR	Loading response
MS	Midstance
TS	Terminal stance
PSw	Pre-swing
SW	Swing
ReLU	Rectified linear unit
FC	Fully connected
KNN	K nearest neighbours
DT	Decision tree
NB	Naive Bayes
LDA	Linear Discriminant Analysis

## Appendix A

**Table A1:** Gait phase recognition accuracy for the deep convolutional neural networks (DCNN) using the signals of separate accelerometer, gyroscope, magnetometer and all above.

Signals	Overall	LR	MS	TS	PSw	Sw
Acc	0.956	0.951	0.938	0.903	0.950	0.990
Ang Vel	0.963	0.965	0.939	0.933	0.963	0.990
Mag	0.951	0.954	0.931	0.889	0.923	0.993
All	0.971	0.973	0.951	0.959	0.954	0.994
